# Charles Bonnet Syndrome as Sequelae of Occipital Lobe Infarct With Hemorrhagic Conversion: A Case Report

**DOI:** 10.7759/cureus.50472

**Published:** 2023-12-13

**Authors:** Alfeo Julius Sy, Diane Charleen Gochioco

**Affiliations:** 1 Department of Clinical Neurosciences, University of the East Ramon Magsaysay Memorial Medical Center, Quezon City, PHL

**Keywords:** neuroophthalmology, neuropsychiatry, visual hallucination, hemorrhagic infarct, charles bonnet syndrome

## Abstract

Charles Bonnet syndrome occurs in the setting of visual impairment with subsequent complex and repetitive visual hallucinations confined in the area of visual loss, with intact cognition and insight. It has been described as a sequelae of ischemic stroke affecting the visual pathway. We report a case of a male presenting with right homonymous hemianopsia secondary to acute left occipital lobe infarct of cardioembolic etiology. He then developed visual hallucinations on the side of the visual loss. MRI showed hemorrhagic conversion of the occipital lobe infarct. Electroencephalogram showed focal and intermittent slowing of the anterior temporal and frontal region. Charles Bonnet syndrome may signify the worsening or progression of a structural lesion affecting the visual pathway, such as hemorrhagic conversion, and warrants prompt and thorough evaluation. Understanding these conditions is crucial for healthcare professionals and caregivers to provide effective support and interventions for those affected.

## Introduction

Charles Bonnet syndrome (CBS) is characterized by the presence of complex visual hallucinations associated with visual impairment in patients with preserved cognitive functions [1]. In Charles Bonnet’s initial report on the disease entity, the core features included visual loss in the setting of normal cognition [2]. This can happen as a result of direct injury anywhere along the visual pathway, including the peripheral visual structures and certain lesions in the central nervous system [3]. The most common condition leading to CBS is ocular disease [4], but rare cases have been reported as sequelae to ischemic stroke [5]. New onset visual hallucinations in the elderly warrant thorough neurologic evaluation as the list of differentials can be varied and all require urgent attention. To our knowledge, this is the first reported case of CBS occurring in the setting of hemorrhagic conversion of an infarct.

## Case presentation

A 73-year-old Asian male presented with right homonymous hemianopsia and episodes of forgetfulness. Cranial imaging revealed acute left occipital lobe infarct of cardioembolic etiology. He was prescribed edoxaban 60 mg once daily and discharged with residual right homonymous hemianopsia. Two weeks after symptom onset, the patient experienced visual hallucinations characterized as silhouettes and shapes of individuals and objects on the side of visual loss, while retaining good insight into the benign nature of these hallucinations. Prior to the events, the patient had good functional status as being independent with activities of daily living including driving, and handling finances. Psychiatric history was unremarkable. Past medical history included hypertension and dyslipidemia.* *Social history was negative for alcohol intake or illicit drug use. He was admitted for further workup and management. Medications at the time of admission were piracetam 1200 mg twice daily, citicoline 500 mg twice daily, and edoxaban 60 mg once daily.

**Figure 1:**
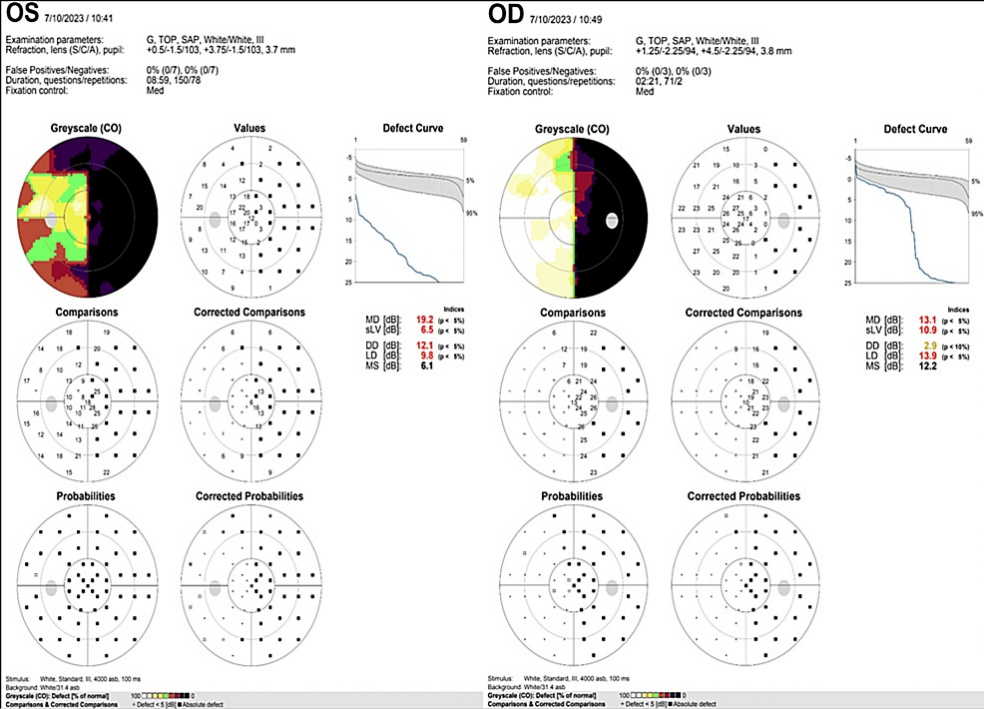
Visual perimetry test. OS (Left Eye) showing right visual field loss (area in black); OD (Right EYE) showing right visual field loss (area in black)

On neurologic examination, the patient reported perceiving the outline of a person in motion, confined to the region of visual loss. He was well aware that these hallucinations were not real and that they did not pose any harm to him. Other pertinent findings include a Montreal Cognitive Assessment - Philippines (MoCA-P) score of 20/30, a visual acuity of 20/70 on the right eye and 20/100 on the left eye, and a right homonymous hemianopsia on the visual confrontation test. A visual perimetry test (Figure 1) was done to confirm visual field loss. Laboratory workup done (Tables 1-2) revealed elevated serum creatinine but was otherwise unremarkable.

**Table 1:** Complete Blood Count

Test	Reference range	Unit	Results
Hemoglobin	140-160	g/L	144
Hematocrit	40-54	%	43
Red Blood Cell	4.5-5.5	x10^12/L	4.5
Mean Corpuscular Hemoglobin Concentration	32-37	%	34
Mean Corpuscular Hemoglobin	27.5-33.2	pg	31.7
Mean Corpuscular Volume	80-94	fL	94
Red Cell Distribution Width	11.0-15.0	%	13.7
White Blood Cell	5.0-10.0	x10^9^/L	6.4
Neutrophils	37-72	%	54
Lymphocytes	20-50	%	39
Eosinophils	0-6	%	7
Platelet	150-440	x10^9^/L	197
MPV	7.5-11.5	fL	9.4

**Table 2:** Metabolic Panel SGOT: serum glutamic-oxaloacetic transaminase; AST: aspartate aminotransferase; SGPT: serum glutamic-pyruvic transaminase; ALT: alanine transaminase; TSH: thyroid stimulating hormone

Test	Reference range	Results
Sodium	135-148 mmol/L	140
Potassium	3.5-5.3 mmol/L	3.8
Blood Urea Nitrogen	2.50-6.70 mmol/L	7.0
Creatinin	49.0-115.0 umol/L	137.4
SGOT (AST)	0.25-0.62 ukat/L	0.28
SGPT (ALT)	0.50-1.09 ukat/L	0.61
TSH	0.25-5 ulU/mL	0.78

**Figure 2:**
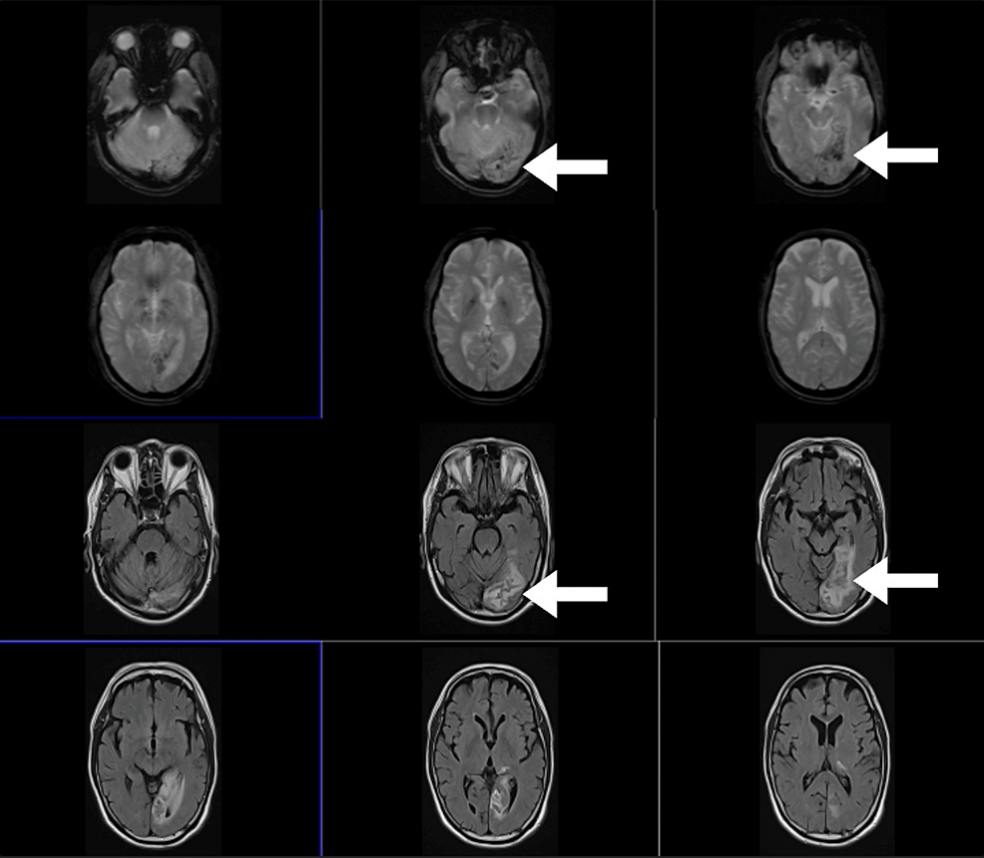
Cranial MRI (GRE – top; T2 FLAIR – bottom) showing a subacute hemorrhagic infarct involving the left posteromedial temporal and left occipital lobes and to a lesser extent the left posterior thalamus GRE: gradient recalled echo; FLAIR: fluid-attenuated inversion recovery

**Figure 3:**
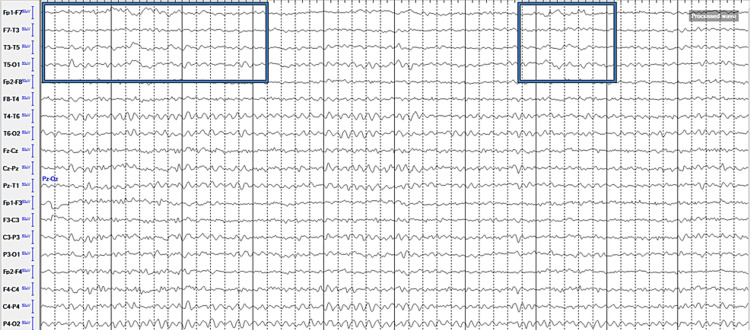
Eelectroencephalogram (EEG). Ten-second epoch tracing with longitudinal bipolar montage and central leads showing focal slowing over the left temporal region (encased in the blue box).

Cranial MRI (Figure 2) showed hemorrhagic conversion of the left occipital lobe infarct. Electroencephalogram (Figure 3) showed focal and intermittent slowing of the anterior temporal and frontal region, with no interictal epileptiform discharges. He was given levetiracetam 500 mg twice daily and the dose of edoxaban was decreased to 30 mg daily to accommodate creatinine clearance. The patient was sent home and followed up after two weeks, with a noted improvement of visual field loss, now limited to the right superior quadrant for both eyes on a visual confrontation test and a decrease in the frequency of the visual hallucinations, now occurring two to three times per week on the side of visual field loss. 

## Discussion

Hallucinations are defined as the perception of an event or object in the absence of an external stimulus [6]. Certain structural lesions of the central nervous system can also cause hallucinations depending on the area affected. Simple hallucinations can occur with occipital lobe lesions, while more complex visual hallucinations are associated with damage to the occipitotemporal or the occipitoparietal visual association area [7], and have also been linked to aberrant activity within visual thalamo-cortical networks [8]. The list of differential diagnoses for elderly patients with visual hallucinations can span over several categories including degenerative diseases such as dementia and Parkinson’s disease [9], vascular disorders such as peduncular hallucinosis [10]), sleep disorders [11], and seizures [12,13]. When other causes of visual hallucinations are reasonably excluded, CBS should be considered [14].

CBS presents as a form of “phantom” vision due to deafferentation of the visual association areas of the cerebral cortex, resulting in the denervated or deafferented cells becoming hyperexcitable [3]. Previously thought of as a rare occurrence, the low number of reported cases has instead been attributed to a difficulty in recognizing the disease entity and often being misdiagnosed as psychosis or early dementia [15]. Patients with CBS retain good insight into the nature of the visual hallucinations, which may also invoke a fear of being misdiagnosed as having an underlying psychiatric or dementing process [1]. Adding to the difficulty in its diagnosis is that there are no specific diagnostic criteria for CBS and there is considerable heterogeneity within published literature [4]. Over the years, there have been several proposed criteria aimed at providing a definition of CBS. The most recent set of criteria, as presented in 2018 by the World Health Organization (WHO) and integrated into the International Classification of Diseases, delineates the diagnostic parameters [16]. These criteria include (i) complex visual hallucinations, (ii) observed in patients experiencing partial or complete loss of vision, and (iii) exclusion of underlying psychiatric symptoms. However, variations in clinicians' interpretation of the quantitative and qualitative dimensions of these criteria lead to differences in diagnostic outcomes.

The prognosis of CBS is benign and will usually resolve within 18 months after the underlying vision deficit improves or is corrected [15]. However, some patients may have persistent release hallucinations, which may cause them significant distress. In such cases, pharmacologic intervention may be warranted. There is no specific or recommended treatment for CBS. Data published on trials of pharmacologic agents including atypical antipsychotics, cholinesterase inhibitors, antiepileptic drugs, and antidepressants have yielded inconsistent results and are mostly case reports or small case series [4,17].

In the setting of a destructive lesion in the occipital lobe, the patient in the current case was started on levetiracetam 500 mg twice daily. Levetiracetam reduces neuronal excitability by its action on the GABAergic system [18]. Some case reports have also reported effective treatment with levetiracetam for CBS [19]. On the patient's follow-up consult, the visual field loss was seen to be limited to a bilateral right superior quadrantanopia on the visual confrontation test, with the frequency of the visual hallucinations down to two to three times per week.

## Conclusions

To the best of our knowledge, this is the first reported case of CBS resulting from hemorrhagic conversion of a subacute occipital lobe infarct. It is important to determine if the new onset visual hallucinations are caused by the preexisting structural lesion, or may be indicative of a change in status such as hemorrhagic conversion. Both CBS and hemorrhagic infarcts represent unique challenges in the realm of neurological and visual health. Understanding these conditions is crucial for healthcare professionals and caregivers to provide effective support and interventions for those affected.
